# Can a nudge keep you warm? Using nudges to reduce excess winter deaths: insight from the Keeping Warm in Later Life Project (KWILLT)

**DOI:** 10.1093/pubmed/fdt067

**Published:** 2013-07-18

**Authors:** Peter Allmark, Angela M. Tod

**Affiliations:** Health and Social Care Research Centre, Sheffield Hallam University, 32 Collegiate Crescent, Sheffield S10 2BP, UK

**Keywords:** affordable warmth, behavioural economics, excess winter deaths, fuel poverty, nudge, social determinants

## Abstract

Nudges are interventions that aim to change people's behaviour through changing the environment in which they choose rather than appealing to their reasoning. Nudges have been proposed as of possible use in relation to health-related behaviour. However, nudges have been criticized as ethically dubious because they bypass peoples reasoning and (anyway) are of little help in relation to affecting ill-health that results from social determinants, such as poverty. Reducing the rate of excess winter deaths (EWDs) is a public health priority; however, EWD seems clearly to be socially determined such that nudges arguably have little role. This article defends two claims: (i) nudges could have a place in tackling even the heavily socially determined problem of EWD. We draw on evidence from an empirical study, the Keeping Warm in Later Life Project (KWILLT), to argue that in some cases the risk of cold is within the person’s control to some extent such that environmental modifications to influence behaviour such as nudges are possible. (ii) Some uses of behavioural insights in the form of nudges are acceptable, including some in the area of EWD. We suggest a question-based framework by which to judge the ethical acceptability of nudges.

## Introduction

The Excess Winter Death rate (EWD) is the excess of deaths in winter compared with non-winter deaths. In 2010/11 there were an estimated 25 700 EWDs in England and Wales, mostly amongst the elderly.^1^ In the Department of Health's recent *Public Health Outcomes Framework for England 2012–16,* reducing EWD is one of the indicators for success in achieving the goal of preventing premature mortality.^2^ Fuel poverty is said to exist where a household needs to spend more than 10% of its income on fuel to maintain satisfactory heating levels; it is estimated that there are 3.2 million fuel-poor households in England;^3^ many members are particularly vulnerable to the cold, physically and mentally.

How might public health professionals meet the goal of reducing EWD? The current UK Government has made numerous positive references to the approach known as ‘nudge’, that is, the use of insights from behavioural psychology to change people's behaviour, in particular, where that behaviour arises from irrational or non-rational processes rather than people's reasoning.^4^ An example might be where someone picks an unhealthy food option having decided not to (perhaps because the unhealthy option was more prominently displayed). However, the Department of Health's 2011 *Cold Weather Plan* makes little mention of this approach.^5^ In addition, the scoping document on EWD from the National Institute for Health and Care Excellence (NICE) makes no reference to behavioural insight or nudge, preferring instead the language of rational persuasion.^6^ In some ways this seems right; an approach focused on behaviour change through management of the choice architecture seems particularly unsuited to tackling EWD, which seems primarily a problem of poverty and housing structure rather than behaviour. Furthermore, the ethics of using nudges in health policy has been criticized as against a principle of respecting people's autonomy, particularly in American literature.^7,8^

This article has two aims. The first is to show that the use of behavioural insights (via so-called nudges) could contribute to public health policy in the apparently unlikely area of EWDs as part of a portfolio of policy. To this end we draw upon selected findings from the Keeping Warm in Later Life project (KWILLT). The second aim is to develop the framework for judging the ethics of nudges suggested in a Parliamentary committee report. The report suggests two questions by which to judge nudges: first, is the nudge visible and, secondly, is it proportionate? To these we suggest three further questions. First, is the end sought through the nudge unequivocally shared in the population; secondly, is it necessary to make a decision to alter the environment required at the time; and thirdly, will the nudge affect health inequality?

## KWILLT

The KWILLT was commissioned by the National Institute for Health Research under its Research for Patient Benefit Programme (grant reference number PB-PG-0408-16041). It aimed to, first, examine the knowledge, beliefs and values of older people regarding keeping warm at home and, secondly, identify the barriers that prevent them accessing help in keeping warm. KWILLT was conducted in Rotherham, an area with high rates of fuel poverty. Ethical approval was granted by East Leeds NHS Research Ethics Committee. This study method and results are described more fully elsewhere.^9^ The research data presented here are previously unpublished except one, which is indicated by a reference to the other paper.

The KWILLT researchers took hourly temperature measurements in people's homes for 1 week prior to collecting qualitative data from interviews with older people. There were also three focus groups at day centres or community groups; participants in these also included carers of the older people. Other than the carers, the participants were people ≥55 years old; although this seems fairly young for the category ‘older people’ it was justified because of the early onset of chronic disease in this deprived population. The participants included people who lived in relatively prosperous parts of the town but who may be asset rich (living in a large property) but cash poor.

Fuel poverty is said to be due to three factors^10^: fuel prices, household income and energy efficiency of the building. The following examples from the KWILLT interviews illustrate each factor:

(i) Fuel prices

‘I keep tabs on all my bills and all my doings. You've got to have a programme, you know what I mean. You've got to be on a budget ... know what's coming on … I've managed to pay my bills but I do have really expensive bills'.

(ii) Household income

‘I get, what is it, £61 a week and out of that I have to put so much towards rent, pay for heating, you know, gas, electric, my water'.

‘I'm not a very wealthy woman; I've just got a bit of pension and one thing and another and I can cope’.

(iii) Energy efficiency

‘I have a big problem with this exterior wall which is a complete mess. The damp's coming through, you cannot decorate it because it just furs up and everything'.

‘It is a very badly insulated house – I'm aware of that'.

And from a staff participant in the study,

'old people living in their own accommodation or private rented housing … You've still got housing that's not been upgraded because people haven't got the money'.

**Fig. 1:**
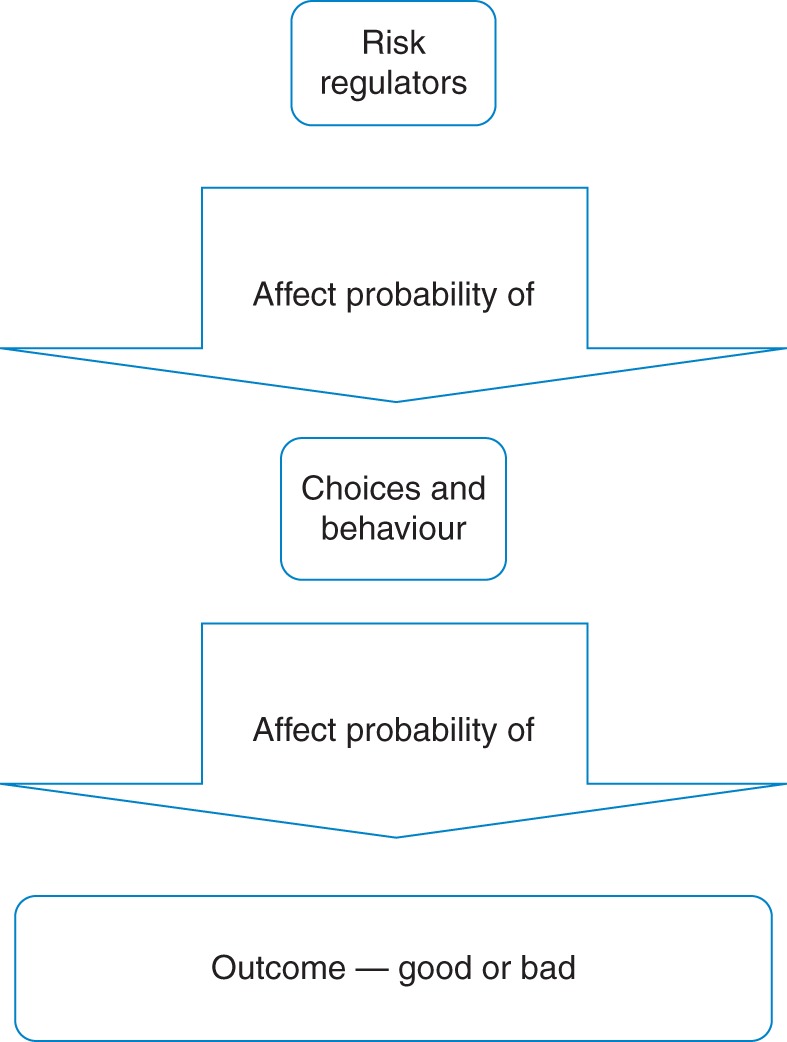
A simple model of risk regulator link to outcome.

**Fig. 2:**
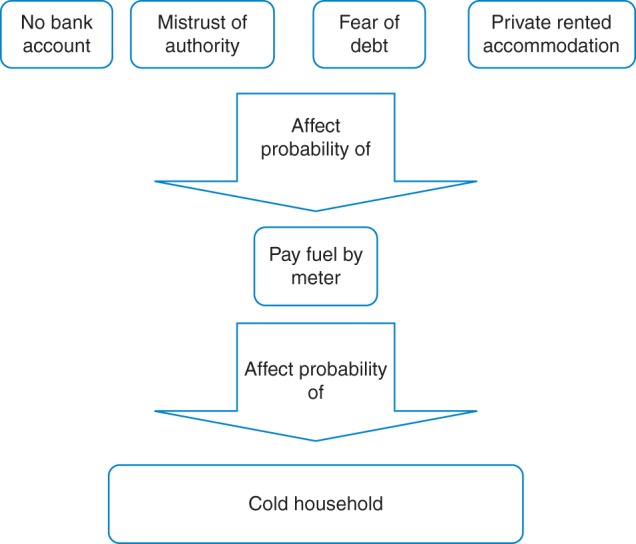
A link between risk regulators and use of pre-payment meters.

In cases such as these the cost of achieving comfortable heat levels is totally prohibitive. In other cases, a low income of itself would not necessarily result in a cold household. However, the addition of further risk regulators increased the likelihood. Risk regulators are factors in the social and physical environment that regulate the probability of a risk manifesting itself.^11^ This is illustrated in Figs 1 and 2.

For example, some participants had important information deficits that compounded the immediate causes of a cold house. Some did not know how to operate their heating systems; even those who understood the system could not necessarily operate it.

‘I just don't know why controls have to be hidden behind a control panel that is fiddly and awkward to access, then it's shaded by everything so it's difficult to see, the numbers are small, the knobs are small. Bring the knobs onto the front, big clear knobs with big clear symbols'.

Another concern arose because central heating systems are veiled; it is not obvious whether they are on or off, or how much they are costing. With visible, stand-alone heaters, this problem does not arise, even though they are, in fact, less efficient and more expensive.

A further information deficit related to financial products. In some cases people had no bank account, using cash only. This closed off the choice of paying by direct debit and thereby receiving the cheapest tariff. Others did not understand or trust paying by direct debit, preferring instead to pay by pre-payment meter which enabled them to know how much they were spending. Again, fear of debt played a part in this choice.

The cultural environment contributed further risk regulators. In previous work by some of the current authors, we noted that people in the former coal mining district of Rotherham often valued independence and were stoic in the face of difficulty.^12^ Many older people interviewed in KWILLT were similarly tough and would refer back to the poverty and adversity of their youth,

‘Oh yes, because I was trained to be frugal, it was part of my upbringing'^9^ [p. 8]

‘ … in the winter my mum used to take the trays out of the oven … wrap them in a blanket and put them in bed for us. I can't get into a warm bed. It has to be cold for me to get into … not too warm, that's unhealthy isn't it?’

Other risk regulators were social isolation, fear of debt, inadequately maintained homes, fear of asking the property owner to repair or increase the energy efficiency of a property in case he increases the rent and mistrust of organizations such as banks or fuel companies.

Thus, each cold household was the result of processes in which differing risk regulators played a part. There were some older people who were not in fuel poverty but nonetheless lived in cold houses because of, for example, fear of debt or inability to work the heating system. Furthermore, economic poverty did not always equate to fuel poverty; those living in social housing are relatively well placed in this regard. Others lived in houses where one room was too hot and the remainder too cold, or where temperatures fluctuated wildly, or were dependent on family members who themselves had information deficits, and so on. It follows that measures to tackle cold and EWD must operate in a complex environment. How might nudge policies contribute?

### The effectiveness of using nudges to reduce EWD

The idea of using nudges in health policy was introduced to governments and the wider public through the eponymous book.^13^ This is concerned primarily with how people make choices in the market, in particular, why they don't always choose rationally as would be predicted by conventional economic theory. The authors argue that people err but do so predictably, for example, with a bias to the *status quo*, or to following the herd. Those working in markets exploit this by manipulating the environment in which people choose. Thus, the preference for following the herd can be used by giving out free packets of cigarettes to attractive young students in the knowledge that paying customers will follow this lead. The authors of *Nudge* argue that it is possible to design choice environments so that people are nudged towards good decisions where they are in danger of making poor ones because of irrational or non-rational elements in their decision-making. Health decisions often have characteristics associated with error; for example, the pleasures of excess are immediate, its costs deferred. As such, public health seems to be an area where the insights of *Nudge* might have a role. There are several behaviours that increase the probability of a cold household some of which are irrational or non-rational in ways that open up the possibility of nudging. Continuing with the use of pre-payment meters as an example, we can develop the simple behaviour model presented earlier.

In this example, one of the risk regulators can be overriding. If people live in private rented accommodation where fuel is supplied by pre-payment meter and they cannot afford to move home then their behaviour is totally constrained; no amount of nudging can help them choose cheaper fuel tariffs. Where that is the case, action needs to be focused at the level of the property owner or above. Similarly, those who are refused bank accounts are not in a position to choose direct debits. However, there are people who are not so constrained but choose to stick with pre-payment meters; they may also operate primarily with cash and choose not to have a bank account. For these people the puissant risk regulators might be fear of debt or mistrust of banks, both factors that seem modifiable. One example was the Child Trust Fund introduced by the Labour Government in the UK in January 2005. This gave people a stake in the system through bank accounts (which many did not previously have) rather than simply one-off payments or stamps; in this way, people were nudged towards using bank accounts.^14^

Let us present a second example. We noted earlier that the design of the heating system could make difficult its use by older people even when they understood it. These problems could be overcome by making the controls accessible and manipulable. It would help also if there were indicative temperature settings on the dial and thermometers in some rooms of the house. For those concerned with fuel usage, smart meters that show the current level of use can help in decision-making about central heating versus stand-alone heaters; having said that, the sight of a meter ticking away could scare people into turning off their heating, especially the fuel poor; like most health interventions, nudges need to be fully evaluated for bad as well as good effects.

The KWILLT study did not test these interventions and as such we present them as hypotheses rather than recommendations. The key point is that nudges could be considered wherever there are risk regulators for behaviour resulting in a cold household that are amenable to change at the individual level. Other risk regulators are strongly constraining over behaviour and thus less amenable to change. It is unlikely that a nudge alone can keep you warm; however, it could help as part of a package of measures. This conclusion concurs with that of the House of Lords Science and Technology Select Committee which published a report on *Behaviour Change* in July 2011:^15^

“ … the most effective means of changing behaviour at the population level was a package of different types of interventions” (para 5.6, p. 33)

### The ethics of using nudges to reduce EWD

The Lords Committee went on to consider the ethics of nudge and set two questions by which to judge the acceptability of a nudge-style intervention. Let us take the hypothetical nudges presented above and consider them in the light of these questions.

- Is it visible in principle? Supermarkets are designed to increase the chance customers will spend; for example, the smell of coffee or baking is introduced into the environment. Although they do not usually notice this, it is not hidden. In the same way, public spaces might be designed to encourage the use of steps for exercise. In contrast, the use of subliminal advertising would not be visible in principle, and this would count against its use. The interventions we have proposed as nudges towards warm-household choices, such as removal of pre-payment meters or placement of room thermometers, are visible in principle.
- Is it proportionate? Here the consideration involves setting the likely amount of harm avoided by the intervention against the costs and harms of the intervention. Answering the question requires in part the work of a health economist but an intervention such as introducing an easy-to-use control panel for a boiler seems likely to be proportionate.
  To these two questions we would add a further three.
- Is the end unequivocal or disputed? The ends sought through some nudges are unequivocal; no reasonable person would prefer environments in which, say, they were more likely to insert their credit card in the wrong way, or forget to turn off the gas when leaving home. Where this is so, it counts in favour of the nudge. Other ends are disputed. Some who smoke, drink or overeat might object to being manipulated towards not doing so. Other ends may be highly disputed; it seems unlikely that all young people would value the avoidance of drug taking, binge drinking and unsafe sex. The more disputed the ends, the less justified the nudge. In relation to cold households, the end would seem at the undisputed end of the spectrum; most people (but not all) would prefer not to live in cold houses and health over illness.
- Is choice-architectural design required? Doors must have handles; pension schemes must have default contribution levels; supermarkets have to put their shelves in some order; organ donation schemes have to be opt-out or opt-in. In contrast, there is no requirement to have posters informing youngsters that drug-taking is a minority pursuit, or that binge drinking exposes you to danger and ridicule. Where choice-architectural design is required it seems reasonable that the design would favour choices all or most people would prefer to make. Where there is no immediate need to change choice architecture this would seem to require a slightly higher level of justification. For example, building new housing that is naturally warm seems perfectly acceptable whilst insulating the house of someone who stoically prefers to be cold does not.
- Will the nudge result in intervention-generated inequality? Interventions that improve public health may also increase health inequality. Typically, well off and educated people gain most from, for example, health education initiatives. Nudge is probably less prone to this problem. It does not seek to change people's behaviour by targeting them directly with, for example, health information. Rather, it involves fashioning the environment in ways that predictably alter choice. Further, it can be targeted towards populations of concern. As such, well targeted nudges could reduce rather than generate inequality. Unfortunately, the forthcoming ‘Green Deal’ in the UK may turn out to be a counter-example. This uses a type of nudge called hyperbolic discounting; people will be offered a loan to insulate their homes which is to be paid back from the resulting savings on bills.^16^ Government advisers have warned of low uptake of this scheme and it seems likely that the most vulnerable to EWD will be the least likely to take it up.^12^ The same or similar deal repackaged without reference to loans and payback, and targeted specifically at the most vulnerable might stand a better chance of success.

## Conclusion

One problem with nudge is that it focuses on individuals rather than on the social determinants of health; it is thus blind to considerations of social justice and to important causes of ill-health that are unrelated to individual choice.^17^ An elderly person in fuel poverty who keeps the heating low or off chooses to do so in a sense; but it is absurd to view her death from EWD as akin to that of a dangerous-sports participant. Any attempt to reduce the elderly person's risk of EWD requires not only that we change the environment in ways that nudge her to choose otherwise; we need also to change it in ways that enable her to choose otherwise. Nudge alone cannot do this. But it has a place in the toolkit.

## Funding

This paper presents independent research commissioned by the National Institute for Health Research (NIHR) under its Research for Patient Benefit (RfPB) Programme (grant reference number PB-PG-0408-16041). The views expressed are those of the authors and not necessarily those of the NHS, the NIHR or the Department of Health. The authors are grateful for the support of the NIHR Collaboration for Leadership in Applied Health Research and Care for South Yorkshire (CLAHRC SY). KWILLT was an adopted project of the Health Inequalities Theme of the CLAHRC SY. Funding to pay the Open Access publication charges for this article was provided by the NIHR Collaboration for Leadership in Applied Health Research and Care for South Yorkshire (CLAHRC SY).
